# Prevalence and spatial distribution of *Theileria parva* in cattle under crop-livestock farming systems in Tororo District, Eastern Uganda

**DOI:** 10.1186/1756-3305-7-91

**Published:** 2014-03-03

**Authors:** Dennis Muhanguzi, Kim Picozzi, Jan Hatendorf, Michael Thrusfield, Susan Christina Welburn, John David Kabasa, Charles Waiswa

**Affiliations:** 1 College of Veterinary Medicine Animal Resources and Biosecurity, Makerere University, P.O. Box 7062, Kampala, Uganda; 2 Centre for Infectious Diseases, School of Biomedical Sciences, College of Medicine and Veterinary Medicine, The University of Edinburgh, Chancellor’s Building, 49 Little France Crescent, Edinburgh EH16 4SB, UK; 3 Swiss Tropical and Public Health Institute, Department of Public Health and Epidemiology, Socinstrasse 57, CH-4002 Basel, Switzerland; 4 Royal (Dick) School of Veterinary Studies, The University of Edinburgh, Edinburgh EH25 9RG, UK

**Keywords:** East Coast fever, Prevalence, p104-based PCR, *Theileria parva*, Tororo District

## Abstract

**Background:**

Tick-borne diseases (TBDs) present a major economic burden to communities across East Africa. Farmers in East Africa must use acaracides to target ticks and prevent transmission of tick-borne diseases such as anaplasmosis, babesiosis, cowdriosis and theileriosis; the major causes of cattle mortality and morbidity. The costs of controlling East Coast Fever (ECF), caused by *Theileria parva,* in Uganda are significant and measures taken to control ticks, to be cost-effective, should take into account the burden of disease. The aim of the present work was to estimate the burden presented by *T. parva* and its spatial distribution in a crop-livestock production system in Eastern Uganda.

**Methods:**

A cross sectional study was carried out to determine the prevalence and spatial distribution of *T. parva* in Tororo District, Uganda. Blood samples were taken from all cattle (n: 2,658) in 22 randomly selected villages across Tororo District from September to December 2011. Samples were analysed by PCR and *T. parva* prevalence and spatial distribution determined.

**Results:**

The overall prevalence of *T. parva* was found to be 5.3%. Herd level prevalence ranged from 0% to 21% with majority of the infections located in the North, North-Eastern and South-Eastern parts of Tororo District. No statistically significant differences in risk of infection were found between age classes, sex and cattle breed.

**Conclusions:**

*T. parva* infection is widely distributed in Tororo District, Uganda. The prevalence and distribution of *T. parva* is most likely determined by spatial distribution of *R. appendiculatus*, restricted grazing of calves and preferential tick control targeting draft animals.

## Background

Tick-borne diseases (TBDs) - anaplasmosis, babesiosis, cowdriosis and theileriosis - cause significant economic losses to the livestock sector in tropical and subtropical regions of the world [1-3]. In Tanzania the annual losses to the livestock sector from TBD have been calculated as US$ 364 million, including mortality of 1.3 million cattle. Theileriosis was estimated to account for 70% of these losses while anaplasmosis, babesiosis and cowdriosis accounted for the remaining 30% [4]. Theileriosis caused by *Theileria parva*, a protozoan parasite transmitted by the brown ear tick (*Rhipicephalus appendiculatus*), infects and transforms lymphocytes, causing a severe and often fatal lymphocytosis called East Coast Fever (ECF) [5]. East Coast fever results in immuno-depression, secondary bacterial infection of the upper respiratory tract, fever and anorexia often leading to death if not treated within three weeks [5-7].

Across Eastern Uganda, cattle are kept under a traditional management system of communal grazing or tethering [8,9]. Cattle are therefore continuously exposed to ticks and thus *T. parva* and other tick-borne infections with some of them progressing to clinical disease (for example ECF). This presents both benefits and risks with regard to TBD epidemiology and in particular to ECF. In the case of anaplasmosis and babesiosis, solid maternal immunity has previously been demonstrated and infection and recovery of calves reduces chances for infection and progression to clinical disease as adults [10,11]. As such, adult cattle that are infected as calves, recover from such infections with the help of maternal immunity and are very resistant to re-infection as adults. This epidemiological situation is called endemic (enzootic) stability and is known to be absolute for both anaplasmosis and babesiosis [10,12]. In case of *T. parva,* which is known to be more virulent than both *Anaplasma* and *Babesia* species, all cattle above 6 months of age are at equal risk of infection with *T. parva* and progression to clinical disease (ECF) [12,13]. East coast fever is more severe than both babesiosis and anaplasmosis with mortality rates varying from 10%-20% in susceptible calves to over 90% in susceptible adults [12-14]. Consequently, very high morbidity and mortality rates due to ECF are expected in areas such as Eastern Uganda, where relatively low transmission rates of *T. parva* have previously been recorded in lowland grazing areas, a topographic situation that typifies Tororo District [15,16]. Conversely, low morbidities and mortalities are expected in highland areas where high transmission and seroconversion at a very young age is expected [15,16]. Even in areas of very high *T. parva* transmission, considerable calf morbidities and mortalities have been recorded [13]. These epidemiological observations indicate that ECF remains an important impediment to livestock production in both areas of low and high transmission rates [13,16].

Farmers in East Africa may use acaracides to target ticks and prevent tick-borne diseases but in Tororo District over 70% of the farmers do not use any acaracides for tick control but rather occasionally hand pick ticks to reduce their populations on cattle [17]. Control options need to be designed that maintain the delicate balance of endemic stability. To examine the extent to which *T. parva* (ECF) presents a problem to the small-scale cattle production systems in Eastern Uganda, a cross sectional study was carried out to determine both the prevalence and spatial distribution of *T. parva* across Tororo District. This information is key for guiding and prioritising TBD control efforts and the integration of such control efforts with those of other economically important livestock vector-borne diseases that occur alongside TBDs in this area, such as trypanosomiasis.

## Methods

### Study area

**Figure 1:**
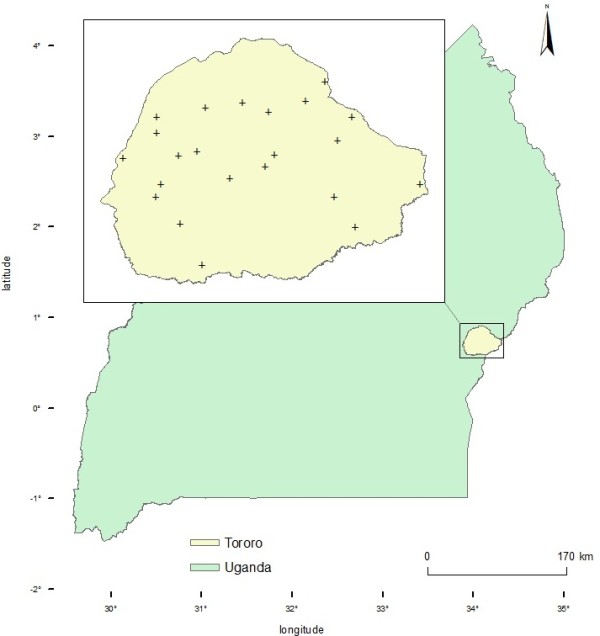
Geographical location of Tororo District (+ sample sites).

The study was carried out in Tororo district (Figure 1) between September and December 2011. The location, farming system, climate and vegetation of the study area have been previously described [8]. At the time of the study, Tororo District had an estimated cattle population of 37,300 [18].

### Study design, sampling and sample size

A complete sampling frame of all villages and their geo-references was obtained from The Coordinating office for Control of Tsetse and Trypanosomiasis in Uganda (COCTU) and verified at the District lands and planning offices. To estimate the prevalence of *T. parva* infections in cattle under traditional farming systems in Tororo District, 22 villages were randomly selected. Sample size was determined assuming a mean cattle population of 93 animals per village [18], anticipated prevalence of AAT of 30% [19], the precision of the sample estimate (one half-length of the 95% confidence interval) of 5%-points and an intra-cluster correlation coefficient (ICC) of 0.15. The ICC estimate was based on reported rates of homogeneity (rho) for vector-borne diseases, noting high variability [20,21].

### Cattle blood sample collection

Blood samples were taken from all cattle in each of the 22 villages in Tororo District. Approximately 125 µl of blood was drawn from the middle ear vein using a capillary tube. Samples were applied to Whatman FTA cards (Whatman Bioscience, Cambridge, UK), avoiding cross contamination [22]. Samples were air-dried at ambient temperature, labelled by the animal ear tag number, names of the parish, sub county, county of Tororo District. Samples were packed in foil pouches with a silica gel desiccant prior to analysis. A temporary marker was used as a means of ensuring that all cattle in the village were sampled, and that each animal was sampled only once.

### DNA extraction

DNA was extracted and eluted from FTA sample discs according to Becker *et al*. [23]. Briefly, each FTA card was placed on a supporting base (Whatman Bio-Sciences Ltd.) and from each of the individual samples; five discs were punched out using a Harris 3.0-mm Micro Punch (Whatman Biosciences Ltd.) and discharged into 1.5 ml Eppendorf tubes. The Micro Punch was cleaned after punching each sample by punching at least the same number of discs from a clean filter paper. A negative control was prepared by cutting the same number of discs from a sterile FTA card and processed together with field samples. Samples were washed twice for 15 min with 1.0 ml FTA Purification Reagent (Whatman) followed by two rinses of 15 min with 1.0 ml TE-1 buffer (10 mM Tris–HCl, 0.1 mM EDTA, pH 8.0). After drying for 45 min at 37°C or at room temperature overnight, the test sample or control discs were boiled at 90°C for 30 min. in DNA Engine Dyad® Cycler -PTC-0221 (Bio-Rad Laboratories Inc.) in 100 µl of 5% (w/v) aqueous suspension of Chelex 100 resin (Sodium form, 50–100 Dry mesh; Bio-Rad Laboratories, Sigma Aldrich, Co., Life sciences, USA). Eluted DNA samples were kept at −20°C for PCR analyses or 4°C if they were to be analyzed within a few days after extraction [22-24].

### Preparation of positive control DNA from *T. parva* sporozoite cell lines

Control DNA was extracted from each of the two *T. parva* frozen sporozoite cell lines (201961TPM and 202094TPM), (kindly provided by Dr. Tim Connelly, of The Roslin Institute, The University of Edinburgh, Easter Bush, Midlothian, EH25 9RG, Scotland, UK) by DNeasy® blood and tissue kit (QIAGEN®, USA). 400ul of the DNA elute were added into a sterile 2 ml micro centrifuge tube and mixed thoroughly by vortexing. 50 µl working stock aliquots were made and kept at 4°C while non-working stock aliquots were stored for future use at −20°C. The extracted DNA was used as control DNA for *T. parva* p104-based PCR [25]. To ensure that the highest quality control DNA was used, the amplicon from the positive control was compared to that from *T. parva* sporozoite cell line DNA (provided by Tanguy Marcotty of the Institute of Tropical Medicine, Antwerp).

### *T. parva* detection by kDa antigen (p104) based PCR

Eluted DNA samples were screened for *T. parva* using a single pair of primers (IL4243; 5-GGC CAA GGT CTC CTT CAG AAT ACG-3 and IL3232; 5-TGG GTG TGT TTC CTC GTC ATC TGC-3) derived from p104 single copy gene [25]. This primer set amplifies a 277 bp fragment of a highly conserved segment of p104 gene making it a very specific and sensitive target for *T. parva* diagnosis. p104-based PCR has a *T. parva* detection level of up to 1.4 parasites/µl of blood compared to 0.4 parasites/µl of blood when nested PCR is used. Conventional p104 based PCR was preferred to the nested PCR format because a large number of samples was handled making nested PCR practically impossible. PCR was performed in 25 µl reaction volume; 20 µl of which were the PCR master mix containing 2.55 µl of 10 ×-reaction buffer (670 mM Tris–HCl pH 8.8, 166 µM (NH4)_2_SO_4_, 4.5% Triton X-100, 2 mg/ml gelatin) (Fisher Biotech), I.0 mM MgCl_2_, 200 µM of each dNTP, 5 µM each of the IL3232 and IL4234 primers, 0.7 U of BioTaq DNA polymerase (Fisher Biotech), 14.55 µl RNase-free water and 5 µl of sample DNA or positive control DNA or negative control eluate. PCR was carried out in a DNA Engine Dyad® Cycler (PTC-0221, Bio-Rad Laboratories Inc.) at cycling conditions including a denaturation step at 95°C for 5 minutes, 30 cycles of denaturation at 94°C for 30s each cycle, annealing at 65°C for 30s, extension at 72°C for 1 minute, final elongation step at 72°C for 5 minutes [26]. PCR products were electrophoresed in 1.5% agarose (Bio Tolls Inc.Japan), stained in GelRed™ (Biotium, Inc., USA) and visualised on a UV transilluminator.

### Data analysis

Raw data were entered into Microsoft Excel™ and analysed using R statistical software v3.0.0. Prevalences and risk factors as well as their confidence intervals were estimated using generalized estimating equation (GEE) models with binary outcome and Logit Link Function to adjust for correlations within communities. *T. parva* spatial analyses were done in ArcMap software environment v10.1 using the spatial analyst extension for Inverse Distance Weighting tool.

### Ethical clearance

This study was cleared by the internal review board of Makerere University College of Veterinary Medicine Animal Resources and Biosecurity and approved by The Uganda National Council for Science and Technology (Registration number HS1336).

## Results

### Demographic characteristics of the study population

2,658 cattle blood samples were collected from the 22 villages between September and December 2011. The mean number of cattle per village was 120 (range 60–200) while the mean number of cattle per household was 4. Almost all the animals belonged to the Boran x short horn Zebu crossbreed. Approximately half of the population were female. The demographic characteristics of the study population are summarised in Table 1.

**Table 1:** **Percentage *T. parva *** **distribution by age, sex and breed (N = 2,658)**

**Population attributes**	**n**	**Positive**	**Prevalence (%)**	**OR**	**95% CI**
**a) Age**					
0-12 months	397	17	4.3	Ref	
13-24 months	579	30	5.2	1.2	0.6-2.6
25-36 months	452	22	4.9	1.1	0.7-2.0
>36 months	1230	64	5.2	1.2	0.7-2.1
**b) Sex**					
Female	1393	67	4.8	Ref	
Male	1069	58	5.4	1.1	0.8-1.5
Neutered	196	8	4.1	0.8	0.4-1.8
**c) Breed**					
Boran × African short horn Zebu (Nkedi)	2570	129	5.0	Ref	
Boran × Holstein Friesian	44	2	4.5	0.9	0.3-2.8
African short horn Zebu (Nkedi)	44	2	4.5	0.9	0.2-3.8

### Prevalence of *T. parva* by age, sex and breed

The distribution of *T. parva* infections stratified by age, sex and breed of cattle is summarised in Table 1. Cattle older than one year of age and males were associated with a slightly higher risk of infection. However, the observed odds ratios were moderate and the confidence intervals included unity indicating no statistically significant difference. There was no difference in the probability of infection with *T. parva* in cattle of different breeds (OR = 0.9; 95% CI; 0.2-3.8).

### Spatial distribution of *T. parva* in Tororo District

**Figure 2:**
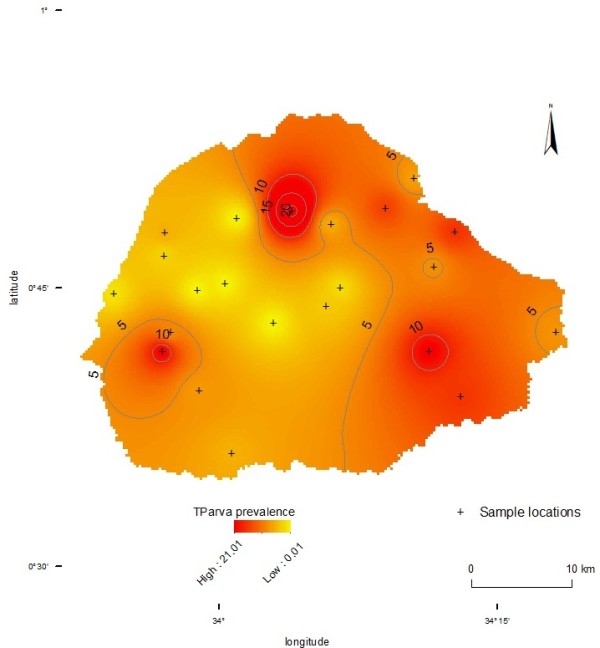
**Prevalence of *T. parva *in Tororo District; September to December 2011.** Shows the spatial distribution of *T. parva* in Tororo district between September and December 2011 interpolated using 22 point prevalences in the 22 study villages to create a district wide spatial effect. An inverse distance weighted interpolation on the analyst extension of ArcMap 10.1 was used to generate a continuous *T. parva* prevalence map on a red colour ramp. Parameters were set so that for each pixel in the continuous raster an average prevalence was calculated based on all prevalence values at village level. Being a weighted average, the weight is higher for villages near the pixel and lower for more distant villages. A default exponent value of 2 was chosen such that each pixel value was taken to be the sum of observed prevalence values, which were first divided by the squared distance between villages. The result was then divided by the number of observations and multiplied by the sum of distances to generate a continuous *T. parva* prevalence map over the 22 individual point prevalences.

The prevalence of *T. parva* in the 22 villages is summarised in Table 2. The overall prevalence of *T. parva* in cattle in Tororo District was 5.3% (95% CI; 3.5-8.0). The prevalence of *T. parva* at herd (village) level was highly variable ranging from 0.0% - 21.1%. In four of the 22 villages no infection was detected in any animal sampled. The majority of infections were detected in villages in the North, North-Eastern and the South-Eastern parts of the district (Figure 2).

**Table 2:** **Village (Herd) level *T. parva *** **prevalence in Tororo District**

**Village**	**Number sampled**	**Number positive**	**Prevalence (%)**	**95% CI**^**a**^
Alupe B	60	5	8.3	2.8-18.4
Atapara-Kaleu	155	14	9.0	5.0-14.7
Chawolo-Sirongo B	188	8	4.3	1.9-8.2
Dida	100	1	1.0	0.0-5.4
Kadanya	132	6	4.5	1.7-9.6
Kajalau Central & South	64	3	4.7	1.0-13.1
Kasoli A	200	23	11.5	7.4-16.8
Kirewa Zone	132	2	1.5	0.2-5.4
Macharimeri	180	6	3.3	1.2-7.1
Mailombiri/Molo-Akisim	103	4	3.9	1.1-9.7
Mikwana/Kijwala	169	0	0.0	0.0-2.2
Munyinyi-Magelo	164	19	11.6	7.1-17.5
Ngeta A	127	4	3.1	0.9-7.9
Nyabanja zone	139	1	0.7	0.0-4.0
Nyafumba A&B	94	3	3.2	0.7-9.0
Oriyoyi A	124	11	8.9	4.5-15.3
Pabendo (Sere A)	76	0	0.0	0.0-4.7
Pamaraka	80	0	0.0	0.0-4.5
Pasaya	104	2	1.9	0.2-6.8
Rubuleri	91	0	0.0	0.0-4.0
Segero-Ojulai	100	5	5.0	1.6-11.3
Singisi	76	16	21.1	12.5-31.9
**Total/Average**	**2,658**	**133**	**5.3**	**3.5-8.0**

^a^*Binomial exact confidence intervals for individual villages adjusted for within village correlation using Generalized estimating equation (GEE) models for the overall estimate.*

## Discussion

To determine the extent of the problem that *T. parva* presents to cattle rearing in Eastern Uganda, a cross sectional study was undertaken to determine the overall prevalence of *T. parva* and examine the spatial distribution of *T. parva* in traditional farming systems in Tororo District. *T. parva* herd prevalence was highly variable between villages (0% - 21.1%) with most of the infections detected in cattle from the North, North-Eastern and the South-Eastern parts of the district. The observed *T. parva* prevalence is in agreement with that previously observed [6] but the individual herd prevalences reported here using PCR based methods are slightly higher. Whereas the serological methods previously used [6] can determine exposure (current and present) to infection, the p104-based PCR used in this study [25] identified current infections; p104-based PCR also has a higher sensitivity and specificity than ELISA. The p104 PCR used in the current study can detect 1.4 parasites/µl corresponding to blood parasitaemia of 2.8 × 10 − ^5^% [27] and the species specificity of the p104 gene PCR assay approaches 100% [25].

Eastern Uganda exhibits a complex pattern of tick challenge with some villages classified as low tick challenge and others as high-tick challenge villages [6]. Low tick-challenge villages were defined as having an average tick infestation rate from 10-70% while high-tick challenge villages recorded a tick infestation that exhibited narrow variation from 90-100% [6]. The spatial distribution of *T. parva* has been shown to mirror that of the tick challenge. The herd seroprevalence of *T. parva* was previously reported to range from 4-5% in villages with low tick challenge and 4-12% in high tick challenge villages [6]. This divergence was thought to relate to the presence of a high density of anti-tick plants (*Lantana camara* and *Ocimum suave*) in villages with low tick challenge and their absence in villages with high tick challenge [6], among other factors. *O. suave* contains oil extracts that can repel and kill all stages of *R. appendiculatus* ticks while *L. camara* oil extracts have been shown to exhibit insecticidal activities [28,29]. The distribution of these plants together with factors such as the microclimate in different villages and variations in the rate at which tick handpicking (a major tick control method) is undertaken may all contribute to the observed spatial variation in the distribution of *T. parva* in Tororo villages.

Almost all cattle sampled in Tororo District were Boran and African short horn Zebu (Nkedi) hybrids and Nkedi [30]. No statistical significant difference in terms of infection with *T. parva* could be established between the predominant breed and other minority breeds in this area largely due to low numbers of other breeds in the sample. African Short horn Zebu (Nkedi) and other African local breeds are reported to be relatively resistant to ticks and tick-borne hemoparasites infections (as compared to exotic animals) and when exposed to infection show a reduced likelihood of developing clinical disease [12,31].

The observed increase of infection with *T. parva* with cattle age may be explained in two ways: i) Maternal antibodies to *T. parva* wane at about 6 months of age [12,31]. Cattle that are more than one year of age have protection from infection by *T. parva* and the progression to clinical disease from acquired immunity developed as a result of exposure to infection as calves (when they were protected by maternal immunity). Previous reports have shown high rates of seroconversion to *T. parva* in calves between 0–6 months in both low and high tick challenge communities and seroconversion rates that parallel tick challenge levels in older cattle [16]. The present study showed slightly elevated prevalences in older cattle that may relate to the sustained tick challenge these animals received under traditional grazing systems, with over 90% of the cattle exposed to tick challenge [8,9]. However, in villages with low tick challenge, a small younger animal population could have been exposed to *T. parva* and most cattle remained susceptible and would seroconvert on subsequent exposure. This situation is likely to maintain a population of susceptible individuals that could be affected by ECF on exposure to *T. parva* infection.

ii) In traditional grazing systems, calves are confined, fed at home and are not permitted to graze with adults to limits their exposure to ticks [32,33]*.* The only time calves between 0–12 months mix with their dams is at the time of milking when they are used to stimulate let-down of milk. This practice keeps calves highly susceptible to TBDs especially at the point at which their maternal antibodies decline (above 6 months of age) and these animals are likely to seroconvert on exposure to *T. parva* infections with the chance of progression to clinical disease.

The extent to which the scenarios (i) and (ii) are likely to lead to the development or disruption of endemic stability development to ECF and other TBDs in Uganda is unclear. It is usually held that indigenous cattle are exposed to constant tick challenge under traditional grazing systems in the crop-livestock production system in Uganda and are therefore likely to develop endemic stability to TBDs and suffer minimal disease; there is however sufficient spatial and seasonal variation in tick abundance/distribution to question this assumption [6]. The current study has shown a high degree of spatial variation in the distribution of *T. parva* that appears to be related to previously reported spatial tick abundance/distribution in Eastern Uganda [6].

Male cattle were associated with a slightly higher probability of infection with *T. parva* than females and castrates, that may be due to a reduction in activity in the individual associated with castration and a reduction of testosterone levels in castrates [34]. Lower physiological activity in females and castrates is associated with lower grazing activity resulting in a reduced probability of the animal encountering *R. appendiculatus* and therefore exposure to infection with *T. parva*[34]. In Eastern Uganda the male to female cattle ratio is high (0.9) since farmers retain many bulls (whole or neutered) for draft power. This herd structure, dominated by animals above 3 years of age and bulls (whole or neutered), is geared towards developing animal traction [35]. Animal traction is the main source of labour in these crop-livestock agricultural systems with bullocks being used to cultivate more than half of their crop fields. The observed slight reduction in the probability of infection with *T. parva* in castrates may be the result of farmers applying preferential tick control (for example tick hand picking) to these animals since bullocks have more value as they are used to provide draft power.

## Conclusion

The prevalence of *T. parva* infections in cattle in different communities in Tororo is highly variable with over a quarter of the animals being infected with *T. parva* in some villages. The prevalence and distribution of *T. parva* is most likely determined by spatial distribution of *R. appendiculatus*, restricted grazing of calves and preferential tick control in draft animals. There is a need to understand the local epidemiology at village level with regard to endemic stability development or its disruption and how these parameters may be exploited in guiding and prioritising TBD control. Routine application of acaracides to cattle is a cost-effective control method that farmers can adopt for both tsetse and tick control in Uganda [36] and is crucial to the long-term success for control of Rhodesian sleeping sickness in Uganda [37].

## Competing interests

The authors hereby declare no competing interests. The sponsors had no role in the study design, data collection and analysis, decision to publish, or preparation of the manuscript.

## Authors’ contributions

DM, MT, JDK, CW and SCW conceived and designed this study. DM carried out blood sample collection and PCR analysis. DM and JH carried out statistical analysis. All the authors participated in the manuscript write up and final approval.
